# The Use of Vagina–Cervix Length Measurement in Evaluation of Future Reproductive Performance of Sows: A Preliminary Study under Commercial Conditions

**DOI:** 10.3390/ani9040158

**Published:** 2019-04-11

**Authors:** Ryszard Tuz, Tomasz Schwarz, Martyna Małopolska, Jacek Nowicki

**Affiliations:** 1 Department of Swine and Small Animal Breeding, Institute of Animal Sciences, University of Agriculture in Kraków, Al. Mickiewicza 24/28, 30-059 Kraków, Poland; rzschwar@cyf-kr.edu.pl (T.S.); j.nowicki@ur.krakow.pl (J.N.); 2 Department of Pig Breeding, National Research Institute of Animal Production, ul. Krakowska 1, 32-083 Balice n. Kraków, Poland; martyna.malopolska@izoo.krakow.pl

**Keywords:** gilt, litter size, reproductive performance, swine, vagina–cervix length

## Abstract

**Simple Summary:**

Animal production is focused on maximizing profits with simultaneous animal welfare protection. Thus, discovery of a simple, stress-free method for gilt selection would be an indispensable element for production improvement and would give necessary knowledge for producers. Our data showed that vagina–cervix length measurements could be a good, additional tool to predict a sow’s likely future reproduction efficiency. The advantages for this method are simplicity, speed, and no cost; the measurement is made as an element of the standard insemination procedure without additional stress and in a relatively early stage of life. Consequently, immediately obtained information gives high reliability and effectiveness in gilt selection.

**Abstract:**

The length of the distal part of the internal reproductive tract seems to be related to the length and capacity of uterine horns, which is the most important anatomical property influencing litter size in sows. The aim of this study was to evaluate variation in vagina–cervix length (VCL) in gilts and differences in reproductive performance of sows according to VCL. The study was performed in a commercial farm using 221 gilts introduced into the breeding herd. Females were divided into three groups: (S) short (26.0 ± 2.0 cm, *n* = 36), (M) medium (31.3 ± 1.46 cm; *n* = 121), and (L) long VCL (36.0 ± 1.4 cm; *n* = 42) (*p* < 0.01). Mean live weight of gilts did not differ significantly among groups. Mean first litter size significantly varied between groups S (10.47 ± 3.01) and L (11.98 ± 2.32) (*p* = 0.0075) and M (10.67 ± 2.98) and L (*p* = 0.0054), while there was no significant difference between group S and M. Significant advantage (*p* = 0.023) was noted in the number of litters obtained from sows in groups L (4.69 ± 3.14), M (3.67 ± 2.71), and S (3.36 ± 2.40), and thus in total life production of sows (*p* = 0.0054), i.e., the number of piglets born alive. To conclude, the differences in vagina–cervix length in gilts during the first service was associated with significant variability in litter size during the first reproductive cycle, giving an advantage to females with longer VCL. Gilts with longer VCL were culled later and gave significantly more litters. Consequently, their lifetime piglet production was greater than gilts with shorter VCL.

## 1. Introduction

An important factor for economic efficiency in pig production is the reproductive potential of sows, particularly litter size [1]. Physiologically, litter size depends on some indirect parameters in which ovulation rate and uterine capacity are of the largest relevance [2]. Selection of gilts for ovulation rate is relatively easy using USG (ultrasound imaging) or laparoscopy techniques to evaluate the number of corpora lutea (CL) in ovaries after ovulation [3,4]. Unfortunately, unilateral selection for ovulation rate gives a good response due to the number of CL, but has only minor effects on litter size in pigs [5]. The main problem is an increase in early embryo mortality due to limited space in the uterus, which reduces the possibility of implantation and growth [6]. Selection of gilts for uterine capacity as the second physiological reproductive coefficient could be effective in terms of the increase in litter size, however, there is no simple method to evaluate it. Currently, the model of selection for litter size as a single feature is performed in most of breeding programs. The effects of such selection, although better than selection for ovulation rate only, are still unsatisfactory. Performance test evaluation of the uterine capacity in gilts and sows is impossible to perform as a direct assessment. The complicated pattern of uterine horn twisting makes it impossible to use laparoscopic, USG, or TC (Computer Tomography) techniques to reliably evaluate the length of the uterine horns, not to mention its capacity [7]. Therefore, some indirect methods were tested with varying levels of success. The measurement of vagina–cervix length (VCL) in gilts seems to be the most promising method, as it is a simple, cheap, and quick evaluation technique. VCL is highly correlated to the length of the uterine horns: a 1 cm longer VCL means 7–8 cm longer horns [8]. Although there is a lack of information about the correlation between the length of horns and their capacity, there is some evidence that longer horns determine a significantly larger number of live fetuses in the uterus at day 30 of pregnancy [9]. Rillo et al. [10] showed that longer VCLs measured in gilts during the first insemination determined larger litter sizes at the first parturition. However, Tarocco and Kirkwood [11] who found litter size was correlated mainly to gilt age and genotype, without significant relation between age and vaginal length. The same authors speculated that there is a threshold at which some minimum vaginal length in gilts at puberty can determine decreased litter size without the possibility of a further increase [11]. The aim of the current study is to evaluate differentiation in VCL in gilts during the second estrus, as measured by a catheter during insemination, and differences in lifetime reproductive performance of sows according to different vagina–cervix length.

## 2. Materials and Methods

### 2.1. Animals and Experimental Procedures

All experimental actions performed on live animals were in compliance with the EU Directive 2010/63/EU for animal experiments and the Polish law for the care and use of animals (2 August 1997). According to Polish law, ethical approval of research is not imperative if experimental procedures are performed as a normal activity on a farm. The present study was carried out in large commercial unit of 900 sows, located near Krakow in southern Poland (longitude: 19°57′E, latitude: 50°04′N). The experiment utilized 221 Polish Landrace x Polish Large White gilts introduced into the breeding herd at the second heat. Before insemination, animals were housed in group pens of 9–10 gilts per pen and fed, ad libitum, dry fodder mix formulated to contain 13.1 MJ ME and 160 g crude protein according to DLG standards (DLG 2011). Females were inseminated (with spiral tip catheter) twice, using 100 mL/3 × 10^9^ of motile spermatozoa portions. The first insemination was performed in the afternoon at the day of estrus detection; the second, 16–18 h after the first treatment. The depth of catheter penetration during insemination was measured to the nearest 1 mm and defined as vagina–cervix length (VCL). After the second insemination, gilts were weighed and moved into individual pens and fed, restrictively 2.5 kg/d, dry fodder mix containing 12.1 MJ ME (megajoules of metabolic energy) and 130 g crude protein for 28 days until pregnancy detection. Then, they were re-grouped into group pens, 6–7 animals per pen, and still fed restrictively the same dry fodder mix until the 90th day of pregnancy. After the 90th day, the feed was changed to a mix containing 13.1 MJ ME and 160 g crude protein. Then, 5–7 days before the expected date of parturition, gilts were moved into the farrowing house and housed in crates until weaning. They were fed, ad libitum dry fodder mix containing 13.1 MJ ME and 160 g crude protein. After weaning, sows were housed in group pens with 8–9 animals per pen, with continued lactation feeding until insemination. After insemination, all procedures were the same as described above. One hundred ninety-nine gilts from 221 were mated effectively with confirmed pregnancies. Twenty-two gilts with repeated heat were excluded from the analyses. Every sows’ parity before each weaning was evaluated in terms of reproductive performance and females that did not attain a minimum standard of the farm were removed from production (Table 1).

Females after the first insemination and pregnancy detection were divided into three groups according to the result of their VCL measurement: group S—short VCL ≤ 28 cm (*n* = 36); group M—medium VCL 28–33.9 cm (*n* = 121); group L—long VCL ≥ 34 cm (*n* = 42). Ranges of VCL for each group were calculated as 1/3 of the whole range, estimated between 21 and 39 cm and excluding values in which no one female was noted. That is why the key factor of division was not the number of females in the group, and groups were different in this term. 

### 2.2. Assessment of Reproductive Performance

The live weight of all animals as well as VCL measurements were taken during the first insemination. All data that it is possible to obtain during normal commercial production were collected after every parturition and included litter size, liveborn litter size, and stillborn litter size. After removing of the sows from production, the analyses of the whole-life number of litters obtained and total-life number of piglets born were performed, as well as the culling level in subsequent parities within groups.

### 2.3. Statistical Analyses

The experimental unit for statistical analyses was the individual animal. Distribution of data was analyzed using the Kolmogorov–Smirnov test. All data were parametric, with normal distribution. The maximum value of the Kolmogorov–Smirnov D coefficient was 0.147, and was non-significant (*p* > 0.05). Live weight and VCL of gilts during the first insemination, as well as reproductive parameters in consecutive reproductive cycles and total life performance were analyzed and compared between three divided groups of females by one-way ANOVA and Duncan’s Multiply Range test using the Statistica version 12 software (StatSoft^®^ Poland, Krakow, Poland). All results are given as mean ± SD unless otherwise stated. Changes in reproductive performance in subsequent parities were analyzed by one-way ANOVA and the LSD test to compare neighboring parities inside each group. Additional analyses were performed for proportions of litter size according to VCL using the Chi-square test (Brandt and Snedecor formula; [12]). The differences in the number of stillborn piglets among VCL groups were additionally analyzed using ANOVA and Duncan’s Multiply Range test according to three divided categories of litter size: small litters <10 piglets, medium litters 10–14 piglets, and large litters >14 piglets. Pearson correlation coefficients were calculated for VCL and reproductive parameter relations, and for litter size and number of stillborn piglets relations. Proportions of culling rate in subsequent parities were analyzed by Chi-square test (Brandt and Snedecor formula; [12]).

## 3. Results

The mean VCL differed significantly (*p* < 0.01) among specified groups of gilts, although there were no significant differences in mean body weight (Table 1). Mean litter size in primiparous sows of group L was significantly (*p* < 0.01) larger than in groups M and S (Table 2). The productivity peak occurred in parity four for group S and M, while for group L, it occurred in parities six or more. There was no significant difference between M and S groups in litter size in any parity, but both groups differed significantly (*p* < 0.01) with group L in the 1st and >6th parities (Table 2). A significant difference in liveborn litter size occurred between groups in the first parity (*p* < 0.05). In subsequent parities, the differences (*p* < 0.05) were noted between the S and M groups (parity 3), L and S groups (parities 3 and 5), and M and L groups (parities 2 and 5) (Table 3). The course of changes in liveborn litter size according to age of sow in groups M and L was typical and characterized by a gradual increase between parities 1 and 3 or 4 (in group L and M, respectively). After this, the high level of productivity was maintained in group L until the >6th parity, while in group M, a swing between increases and decreases started after the 4th parity. Such a swing of increases and decreases of liveborn litter size was observed from the beginning between subsequent parities from 1 to >6 in group S (Table 3). This effect was not noted in total born litter size in group S, where a gradual increase was observed from parity 1 to 4, and then the course was similar to that observed in group M. The appearance of a large number of stillborn piglets in large litters in group S raised the total number of piglets born (Table 2 and Table 4). A similar effect was observed in group M, while in group L the relation was least visible. The significant difference (*p* < 0.01) between group S and L in mean stillborn litter size was noted in 4th and 6th parities. Moreover, the highest number of stillborn piglets per litter in group S was noted (Table 4).

All values of total lifetime reproductive performance except for the number of stillborn piglets were significantly higher (*p* < 0.01) in group L compared to groups M and S. Group L obtained more piglets per litter and over one parity compared to the other analyzed groups. Whereas, the differences between groups M and S were mostly not significant, the mean whole life liveborn litter size was significantly larger (*p* <0.05) in group M (Table 5).

The largest level of culling of sows after the 1st parity was observed in group S, while after the 2nd parity it was in groups M and L (Table 6). A 60% threshold value of culling rate was achieved first in group S (after the 3rd litter), then in group M (after the 4th litter), and finally in group L (after the 6th litter). A 90% threshold value of culling rate was achieved after the 6th litter in group S, after the 7th litter in group M, and after the 9th litter in group L. In most parities, the culling rate was significantly lower in group L than in groups S and M, whereas after the 6th litter, there was also a significant difference (*p* < 0.05) between groups M and S (Table 6).

The analyses of proportions among litter size according to VCL showed a significantly (*p* < 0.01) lower percentage of small litters in group L in comparison to groups S and M and a significantly (*p* < 0.01) larger percentage of large litters in M group in comparison to S group, and in L group in comparison to groups S and M (Table 7). The percentage of medium litter sizes were similar in every group, however, a significant difference was noted between groups S and M (Table 7).

The analyses of the number of stillborn piglets showed a significant (*p* < 0.01) increase concurrent with increasing litter size in all VCL groups and in total number of analyzed sows (Table 7). The Spearman correlation between litter size and the number of stillborn piglets was 0.38, 0.41, and 0.37 for S, M, and L groups, respectively, and every group was significant (*p* < 0.01; Supplementary Table S2). There were no significant differences among VCL groups in small litters, but in medium litters the number of stillborn piglets was significantly (*p* < 0.01) lower in group L, whereas in large litters the number of stillborn piglets was significantly (*p* < 0.01) larger in S group in comparison to M and L groups. The same relationship was found in the number of mummified fetuses (Table 7). The highest number of mummies were found in group S, which achieved large litters (1.77) in comparison to groups M (0.29) and L (0.35).

The Spearman correlation between VCL and reproductive parameters showed significant (*p* < 0.01) coefficients to the number of litters per sow (r = 0.233); parity 1 live born (r = 0.227) and total born litter size (r = 0.248); mean life live born (r = 0.374) and total born litter size (r = 0.410); and total life number of live piglets (r = 0.292), stillborn piglets (r = 0.184), and number of piglets (r = 0.289; Supplementary Table S1). 

## 4. Discussion

A gilt’s body weight and composition at the first inseminations is recognized as one of the important traits affecting future reproductive performance and longevity of sows due to protection against excessive body-weight losses during the first lactation [13]. Moreover, the gilt’s body growth needs to be in harmony with reproductive tract development to achieve a good fertility rate [14,15,16]. In our study, no significant differences between body weight in gilt groups were found, while changes in the length of external reproductive organs were clearly evident. This could suggest that excessive emphasis on body weight as a decisive feature for admission of females to reproduction is inappropriate. Other authors’ results also showed variability in vagina–cervix length in gilts and sows [17,18]. Interestingly, each pig breed seems to have its own specific range of reproductive tract length. For example, Rillo et al. [10] divided Duroc gilts into three groups based on VCL measurement, where the group with smallest VCL was ≤ 24 cm; medium VCL >24 and <26 cm, and large with VCL >26 cm. Whereas the results of our research, conducted on Landrace x Large White gilts, showed a wider range of VCL: small < 28 cm, medium from 28 to 33.9 cm, and large ≥ 34 cm. Additionally, the comparison of group sizes in both experiments showed a different distribution of animals inside the groups: small were the most numerous, then medium, then large [10], whereas, in our research the most numerous was the medium group, than large, than small. The genetic potential related to a sow’s exploitation seems to be the main reason for differences observed between these experiments. Durocs are a typical terminal sire line emphasizing progeny growth while the Landrace x Large White is a typical dam line with larger productivity in reproduction. However, in spite of the genetic potential, the growth and development of reproductive organs is influenced by many factors such as litter size, the sex ratio of a gilts’ birth litter, nutrition, and growth rates [19,20]. Consequently, great variability of VCL within breed, in the same housing conditions, may occur. Also, this comprehensive impact on gilt body and organ growth and development makes it difficult to predict precisely the future reproduction efficiency of a gilt. One of the limiting factors is uterine capacity, which affects litter size, but the relationships occurring between reproductive tracts are still not fully described and remain unclear. Fertilization rate in pigs is high, reaching 95–100%, but 30–50% of conceptuses are lost in the early stage of gestation [21]. The most important cause of embryo/fetal loses seems to be limited space inside uterus [22,23,24]. Evidently, some gilts (in the same breed and housing conditions) are more likely to obtain large litters. According to Dybała et al. [14] and Rillo et al. [10], the number of piglets born in a litter is correlated with VCL, thanks to the relationship between VCL and uterine horn length. Based on this theory, the sows with a longer VCL have longer uterine horns and, thus, larger litters in comparison to females with shorter external reproductive tracts from the same breed, age, and body weight. Our study showed significant difference in the first and second parity litter size between gilts with large VCL and two other groups. For the more important measure of live born litter size, an advantage of group L was visible in almost every parity, excluding the 4th parity where the M group slightly exceeded the L group. Total life production is clearly the best for the gilts with the longest VCL; those sows gave more litters, and the average number of piglets is larger both per litter and in total, confirming a theory that based on the number of piglets born alive in the first and second parities, it is possible to predict a sow’s lifetime performance [25]. In groups S and M, a significant increase of litter size was noted until the 4th parity and then showed alternate relevant peaks and nadirs. These results are consistent with research conducted by Freyer [26], where the greatest litter sizes were also reached by sows in their fourth parity. On the other hand, some researchers showed that the number of piglets born per litter increases with sows’ parity, reaching a peak between 2 to 5 parities [27,28]. An important difference between sows from these two groups was that changes in the number of live born piglets in our group M were concurrent to the total born litter size, while in group S a rapid rise of stillborn piglets in parities 3 and 4 caused a relevant discrepancy between total born and live born litter size. That is why the increase in the number of live born piglets among parities 2 and 4 was not significant. Our results support the theory that a large number of piglets per litter can increase not only early embryo mortality but also late fetal mortality [2,29]. However, this relation was clearly visible and statistically significant in sows from each group; in sows with long VCL the increase in number of stillborn piglets occurred much latter. These females also obtained their maximum productivity much later, in >6th parity, and interestingly, differences between neighboring parities were not significant during whole sows’ life. This indicates that the 4th parity was the most productive for sows from small and medium groups causing shortened usability of most of them, while sows from the L group could be useful much longer, maintaining a high level of performance stability, whether live born or total born litter size is analyzed. It is also worth mentioning that even the smallest first liveborn litter size in group L was larger than the largest (4th parity) litter in group S, which indicates and clearly confirms that the size of reproductive organs is a limiting factor for litter size. Consequently, sows from group L showed higher lifetime performance than sows from other groups. Differences between groups L and S, and L and M in mean litter size, liveborn litter size, total number of piglets born, and total number of live born piglets were visible within the herd. This disproportion in reproductive performance inside the herd/breed was also shown by other authors [30]. Lifetime performance is mostly described as total number of liveborn piglets and mean liveborn piglets per parity [31]. The sow’s lifetime productivity is influenced by many factors such as management, health, genotype, housing system, and nutrition. Reproduction failures are the most common reason to cull sows in early parities [30]. Our results showed that 56.3% of all analyzed sows were culled by the 3rd parity. The culling rate of sows was the greatest after the first parity in each of analyzed groups. Interestingly, however, the culling rate was associated with VCL. The lowest level of early culling was noted in group L for analyzed parities, which indicated that stayability and longevity could be related to VCL. Worldwide research results show relationships between litter size, variation in birth weight, and occurrence of stillborn piglets [32,33,34]. Sows that obtain large litters are more likely to have an increased number of stillborn piglets and piglets with low birth weight. Additionally, low birth weight could have a negative impact on future production efficiency and lead to higher mortality in the pre-weaning period [35]. For all groups, distribution of medium size litters was about 60%. The percentage of small litter size was the lowest in group L, and the highest in group S. In contrast, in the case of large litters, group L differed significantly from both S and M groups. These data clearly show that sows with a short VCL are more likely to obtain small litters, while sows from group L tend to obtain large litters. Interestingly, our results give an additional view on litter size and occurrence of stillborn piglets. Despite litter size, the number of stillborn piglets and number of mummified fetuses were the greatest in sows with a short VCL. In large litters, the number of stillborn and mummified fetuses was reached by group S, while there were no differences between groups M and L. Thus, distribution of litter size depended on VCL. Sow reproductive tract and litter size are inseparably connected; a limitation in the size of the organs reduces the litter size or increases the occurrence of stillborn piglets or mummified fetuses.

## 5. Conclusions

In conclusion, our results showed a wide range in reproductive tract development in the same age gilts, independent from body mass. We found the possibility of using catheter penetration depth as a simple method of VCL measurement to distinguish elite gilts with the longest VCL and thus a strong positive impact on future reproductive performance and longevity. Surprisingly, no significant effect of catheter penetration depth on reproductive performance was visible in the comparison of short and medium VCL gilts. Based on this data, the confirmation of the relationship between VCL and reproductive efficiency gives hope for an additional tool for selection of elite gilts (in breeding programs). However, more research is required to corroborate VCL effects in different pure breed and cross breed pigs, using larger numbers of animals, separately for each possible genotype.

## Figures and Tables

**Table 1:** The number of gilts that qualified for analysis (successfully farrowed in the first parity), number of sows in subsequent reproductive cycles, and comparison of mean body weight and vagina–cervix length in the first mating period among gilts in the specified groups.

Item	Experimental Groups VCL Range (cm)						Total 21.0–39.0	
	S 21.0–28.0		M 28.1–33.9		L 34.0–39.0			
Total number of gilts	36		121		42		199	
Number of sows in:								
II parity	25		88		33		146	
III parity	21		64		28		113	
IV parity	13		51		23		87	
V parity	11		38		21		70	
VI parity	7		31		17		55	
VII parity	3		26		13		42	
VIII parity	2		9		8		19	
IX litter	1		8		6		15	
X litter	1		6		4		11	
XI litter	1		1		2		4	
XII litter	0		1		0		1	
Total number of litters	121		444		197		762	
	Mean	SD	Mean	SD	Mean	SD	Mean	SD
Mean VCL (cm)	26.0 ^A^	2.0	31.3 ^B^	1.6	36.0 ^C^	1.4	31.3	3.5
Mean body weight (kg)	136.7	6.4	136.4	4.3	138.1	4.4	136.9	4.6

VCL: vagina–cervix length. Within rows, means denoted by the different letter superscripts differ significantly ^ABC^ *p* < 0.01.

**Table 2:** A comparison of mean litter size among sows in specified groups in subsequent reproductive cycles.

Parity Number	Experimental Groups						Average	
	S		M		L			
	Mean	SD	Mean	SD	Mean	SD	Mean	SD
I	10.47 ^A^	3.01	10.67 ^A,*^	2.98	11.98 ^B^	2.32	10.91 ^**^	2.90
II	11.32 ^a^	3.13	11.39 ^a**^	2.70	12.64 ^b^	2. 69	11.66 ^*^	2.81
III	11.62 ^**^	2.60	12.69	3.24	12.61	3.24	12.47	3.13
IV	13.85 ^*^	2.44	13.47 ^*^	3.82	12.39	3.22	13.24 ^*^	3.50
V	11.45	2.94	11.95	3.50	13.14	2.82	12.23	3.24
VI	13.43 ^**^	1.40	12.97	3.24	12.94	2.49	13.02	2.81
>VI^	10.33 ^A^	3.04	11.92 ^A^	3.17	14.09 ^B^	2.71	12.55	3.23

Within rows, means denoted by different letter superscripts differ significantly: ^AB^ *p* < 0.01; ^ab^ *p* < 0.05. Within columns, the upper neighboring means that differ significantly from the lower means are denoted by asterisks: ** *p* < 0.01; * *p* < 0.05. ^ Due to the low number of sows over VI parity, the data of litter size were pooled to ensure proper statistical analyses.

**Table 3:** A comparison of mean liveborn litter size among sows in specified groups in subsequent reproductive cycles

Parity Number	Experimental Groups						Average	
	S		M		L			
	Mean	SD	Mean	SD	Mean	SD	Mean	SD
I	9.31 ^Aa*^	3.08	9.84 ^Ab*^	3.27	11.07 ^B^	2.23	10.01 ^**^	3.09
II	10.76 ^ab^	2.73	10.63 ^a*^	2.43	11.55 ^b^	2.49	10.86	2.51
III	10.19 ^a^	3.36	11.59 ^b^	3.03	11.86 ^b^	3.18	11.40	3.16
IV	10.92	2.96	11.78	3.64	11.22	2.75	11.51	3.31
V	9.91 ^a^	2.91	10.76 ^a^	3.30	12.14 ^b^	2.94	11.04	3.19
VI	10.86	1.57	11.32 ^*^	2.01	12.12	2.47	11.51	2.12
>VI^	8.63 ^A^	2.83	9.96 ^A^	2.33	11.94 ^B^	2.82	10.55	2.76

Within rows, means denoted by different letter superscripts differ significantly: ^AB^ *p* < 0.01; ^ab^ *p* < 0.05. Within columns, the upper neighboring means that differ significantly from the lower means are denoted by asterisks: ** *p* < 0.01; * *p* < 0.05. ^ Due to the low number of sows over VI parity, the data of litter size were pooled to ensure proper statistical analyses.

**Table 4:** A comparison of mean stillborn litter size among sows in specified groups in subsequent reproductive cycles.

Parity Number	Experimental Groups						Average	
	S		M		L			
	Mean	SD	Mean	SD	Mean	SD	Mean	SD
I	1.17	2.54	0.83	1.82	0.90	1.36	0.90	1.88
II	0.56 ^*^	0.92	0.76	1.30	1.09	1.68	0.80	1.35
III	1.43 ^*^	2.29	1.09	2.09	0.75	1.14	1.07 ^**^	1.94
IV	2.92 ^Aa^	2.50	1.69 ^ABb^	1.87	1.17 ^Bb^	1.44	1.74 ^*^	1.93
V	1.55	1.69	1.18	1.96	1.00	1.41	1.19	1.76
VI	2.57 ^A*^	2.15	1.65 ^AB^	2.29	0.82 ^B^	1.01	1.51	2.01
>VI^	1.50	2.45	1.90	2.02	2.15	2.25	1.96	2.13

Within rows, means denoted by different letter superscripts differ significantly: ^AB^ *p* < 0.01; ^ab^ *p* < 0.05. Within columns, the upper neighboring means that differ significantly from the lower means are denoted by asterisks: ** *p* < 0.01; *p < 0.05. ^ Due to the low number of sows over VI parity, the data of litter size were pooled to ensure proper statistical analyses.

**Table 5:** A comparison of mean and total life production of piglets per sow, among sows in specified groups.

Life Production	Experimental Groups						Total	
	S		M		L			
	Mean	SD	Mean	SD	Mean	SD	Mean	SD
Litters per sow	3.36 ^a^	2.40	3.67 ^a^	2.71	4.69 ^b^	3.14	3.83	2.78
Mean litter size	11.4 ^A^	2.99	11.83 ^A^	3.28	12.79 ^B^	2.81	12.02	3.15
Mean liveborn litter size	10.0 ^Aa^	2.97	10.67 ^Ab^	3.03	11.63 ^B^	2.67	10.82	2.98
Mean stillborn litter size	1.41	2.20	1.16	1.89	1.16	1.61	1.20	1.88
Total number of piglets	38.47 ^A^	30.33	43.41 ^A^	35.62	59.98 ^B^	43.33	46.02	37.10
Total liveborn piglets	33.72 ^A^	25.36	39.15 ^A^	31.16	54.55 ^B^	38.98	41.42	32.67
Total stillborn piglets	4.75	6.46	4.26	6.07	5.43	6.18	4.60	6.15

Within rows, means denoted by different letter superscripts differ significantly: ^AB^ *p* < 0.01; ^ab^ *p* < 0.05.

**Table 6:** A comparison of the culling rate of sows after subsequent farrowings among specified groups, reflected as the number of eliminated sows, percent of the eliminated sows in relation to the initial group size, and the percent increase in relation from litter to litter.

Culling Rate After	Number			%			% increase			Total		
	Experimental Groups			Experimental Groups			Experimental Groups					
	S	M	L	S	M	L	S	M	L	Number	%	% incr.
I litter	11	33	9	30.6	27.3	21.4	-	-	-	53	26.6	-
II litter	4	24	5	11.1	19.8	11.9	41.7 ^ab^	47.1 ^a^	33.3 ^b^	33	16.6	43.2
III litter	8	13	5	22.2 ^a^	10.7 ^b^	11.9 ^ab^	63.9 ^a^	57.9 ^ab^	45.2 ^b^	26	13.1	56.3
IV litter	2	13	2	5.6	10.7	4.8	69.4 ^a^	68.6 ^a^	50.0 ^b^	17	8.5	64.8
V litter	4	7	4	11.1	5.8	9.5	80.6 ^a^	74.4 ^a^	59.5 ^b^	15	7.5	72.4
VI litter	4	5	4	11.1	4.1	9.5	91.7 ^Aa^	78.5 ^ABb^	69.1 ^Bb^	13	6.5	78.9
VII litter	1	17	5	2.8 ^a^	14.1 ^b^	11.9 ^ab^	94.4 ^a^	92.6 ^a^	80.9 ^b^	23	11.6	90.5
VIII litter	1	1	2	2.8	0.8	4.8	97.2 ^a^	93.4 ^a^	85.7 ^b^	4	2.0	92.5
IX litter	0	2	2	0	1.7	4.8	97.2	95.0	90.5	4	2.0	94.5
X litter	0	5	2	0	4.1	4.8	97.2	99.2	95.2	7	3.5	98.0
XI litter	1	0	2	2.8	0	4.8	100.0	99.2	100.0	3	1.5	99.5
XII litter	-	1	-	-	0.8	-	-	100.0	-	1	0.5	100.0

% incr. = % increase. Within rows for the same parameter (% or % incr.), means denoted by different letter superscripts differ significantly: ^AB^ *p* < 0.01; ^ab^ *p* < 0.05.

**Table 7:** Percentage of litters in three categories of litter size depending on VCL, mean (±SD) number of stillborn piglets, and number of mummified fetuses depending on litter size and VCL.

Litter Size	Experimental Group						Total	
	S		M		L			
**Percentage of litters**								
Small (<10)	23.14% ^A^		22.52% ^A^		9.14% ^B^		19.16%	
Medium (10–14)	66.12% ^a^		57.43% ^b^		61.93% ^ab^		59.97%	
Large (>14)	10.74% ^A^		20.05% ^B^		28.93% ^C^		20.87%	
Total	100.00%		100.00%		100.00%		100.00%	
**Number of still born piglets**								
	Mean	SD	Mean	SD	Mean	SD	Mean	SD
Small (<10)	0.36 ^X^	0.87	0.31 ^X^	1.01	0.39 ^X^	0.78	0.33 ^X^	0.95
Medium (10–14)	1.29 ^AY^	1.76	1.00 ^AY^	1.66	0.76 ^BX^	1.38	0.99 ^Y^	1.62
Large (>14)	4.46 ^AZ^	3.71	2.57 ^BZ^	2.43	2.25 ^BY^	1.75	2.61 ^Z^	2.40
Total	1.41	2.20	1.16	1.89	1.16	1.61	1.20	1.88
**Number of mummified fetuses**								
	Mean	SD	Mean	SD	Mean	SD	Mean	SD
Small (<10)	0.14 ^X^	0.45	0.05 ^Xx^	0.22	0.06 ^X^	0.24	0.07 ^X^	0.28
Medium (10–14)	0.53 ^AY^	0.87	0.13 ^BbXy^	0.43	0.05 ^BcX^	0.25	0.18 ^Y^	0.53
Large (>14)	1.77 ^AZ^	1.48	0.29 ^BY^	0.57	0.35 ^BY^	0.74	0.43 ^Z^	0.84
Total	0.57	0.98	0.15	0.43	0.13	0.46	0.21	0.58

Means within row with different superscripts differ (^a,b^, *p* < 0.05, ^AB^ *p* < 0.01). Means within columns with different superscripts differ (^XYZ^ *p* < 0.01; ^xyz^ *p* < 0.05).

## Supplementary Materials

The following are available online at https://www.mdpi.com/2076-2615/9/4/158/s1, Table S1: Summary of correlations between vagina–cervix length (input) and the most important reproductive variables (output) in the analyzed gilts (*n* = 199). Table S2: Summary of correlations between litter size and the number of stillborn piglets for groups of sows divided according to VCL (S—short VCL, M—middle VCL and L—long VCL).
