# Supplementary material for: The Use of Vagina–Cervix Length Measurement in Evaluation of Future Reproductive Performance of Sows: A Preliminary Study under Commercial Conditions
Source: Animals (Basel). 2019 Apr 11;9(4):158. doi: 10.3390/ani9040158 (PMC6523857; doi:10.3390/ani9040158)
Supplement: Supplementary file 1 [file animals-09-00158-s001.pdf]

animals

Supplementary: The Use of Vagina-Cervix Length Measurement in the Valuation of Future Reproductive Performance of Sows: A Preliminary Study in Commercial Conditions

Ryszard Tuz [1,*], Tomasz Schwarz [1], Martyna Małopolska [2] and Jacek Nowicki [1]

[1] Department of Swine and Small Animal Breeding, Institute of Animal Sciences, University of Agriculture in Kraków, Al. Mickiewicza 24/28, 30-059 Kraków, rzschwar@cyf-kr.edu.pl (T.S.), j.nowicki@ur.krakow.pl (J.N.)
[2] Department of Pig Breeding, National Research Institute of Animal Production, ul. Krakowska 1, 32-083 Balice n. Kraków, Poland; martyna.malopolska@izoo.krakow.pl
* Correspondence: rztuz@cyf-kr.edu.pl, Tel.: +48-12-662-40-11

Received: 27 February 2019; Accepted: 2 April 2019; Published: date

Table S1. Summary of correlations between litter size and the number of stillborn piglets for groups of sows divided according to VCL (S—short VCL, M—middle VCL, and L—long VCL).

Input (x) variable	Output (y) variable	Regression equation	*p*-value	r
Litter size	Stillborn litter size (all litters)	y = 0.23x - 1.57	0.000000	0.388
Litter size S	Stillborn litter size (S)	y = 0.28x - 1.77	0.000020	0.377
Litter size M	Stillborn litter size (M)	y = 0.24x - 1.65	0.000000	0.414
Litter size L	Stillborn litter size (L)	y = 0.21x - 1.56	0.000000	0.371

Table S2. Summary of correlations between vagina–cervix length (input) and the most important reproductive variables (output) in the analyzed gilts (*n* = 199).

Input (x) variable	Output (y) variable	Regression equation	*p*-value	r
VCL (cm)	Parity 1 live born litter size	y = 0.20x + 3.77	0.001	0.227
VCL (cm)	Total life live born number of piglets	y = 2.71x + 43.52	0.00003	0.292
VCL (cm)	Mean life live born litter size	y = 0.25x + 2.43	0.00000	0.374
VCL (cm)	Parity 1 stillborn litter size	y = 0.005x + 0.75	0.900	0.009
VCL (cm)	Total life stillborn number of piglets	y = 0.32x – 5.44	0.009	0.184
VCL (cm)	Mean life stillborn litter size	y = 0.02x + 0.54	0.590	0.038
VCL (cm)	Parity 1 total born litter size	y = 0.20x + 4.52	0.0004	0.248
VCL (cm)	Total life number of piglets	y = 3.04x – 49.21	0.00004	0.289
VCL (cm)	Mean life litter size	y = 0.27 + 2.97	0.00000	0.410
VCL (cm)	Total life number of litters	y = 0.18 – 1.93	0.0009	0.233

© 2019 by the authors. Submitted for possible open access publication under the terms and conditions of the Creative Commons Attribution (CC BY) license (http://creativecommons.org/licenses/by/4.0/).
